# No impact of sex on surgical site infections in abdominal surgery: a multi-center study

**DOI:** 10.1007/s00423-022-02691-6

**Published:** 2022-10-10

**Authors:** Simone N. Zwicky, Severin Gloor, Franziska Tschan, Daniel Candinas, Nicolas Demartines, Markus Weber, Guido Beldi

**Affiliations:** 1 Department of Visceral Surgery and Medicine, Inselspital, Bern University Hospital, University of Bern, Bern, Switzerland; 2 Institute for Work and Organizational Psychology, University of Neuchâtel, Neuchâtel, Switzerland; 3 Department of Visceral Surgery, University Hospital Lausanne (CHUV), Lausanne, Switzerland; 4 Department of Surgery, Triemli Hospital, Zurich, Switzerland

**Keywords:** Sex, Surgical site infection, Abdominal surgery

## Abstract

**Objective:**

Male sex is controversially discussed as a risk factor for surgical site infections (SSI). The aim of the present study was to evaluate the impact of sex on SSI in abdominal surgery under elimination of relevant confounders.

**Methods:**

Clinicopathological data of 6603 patients undergoing abdominal surgery from a multi-center prospective database of four Swiss hospitals including patients between 2015 and 2018 were assessed. Patients were stratified according to postoperative SSI and risk factors for SSI were identified using univariate and multivariate analysis.

**Results:**

In 649 of 6603 patients, SSI was reported (9.8%). SSI was significantly associated with reoperation (22.7% vs. 3.4%, *p* < 0.001), increased mortality rate (4.6% vs. 0.9%, *p* < 0.001), and increased rate of length of hospital stay > 75th percentile (57.0% vs. 17.9%, *p* < 0.001). In univariate analysis, male sex was a significant risk factor for SSI (*p* = 0.01). In multivariate analysis including multiple confounders’ such as comorbidities and perioperative factors, there was no association between male sex and risk of SSI (odds ratio (*OR*) 1.1 [*CI* 0.8–1.4]). Independent risk factors for SSI in multivariate analysis were BMI ≥ 30 kg/m^2^ (*OR* 1.8 [*CI* 1.3–2.3]), duration of surgery > 75th percentile (*OR* 2.3 [1.8–2.9]), high contamination level (*OR* 1.3 [1.0–1.6]), laparotomy (*OR* 1.3 [1.0–1.7]), previous laparotomy (*OR* 1.4 [1.1–1.7]), blood transfusion (*OR* 1.7 [1.2–2.4]), cancer (*OR* 1.3 [1.0–1.8] and malnutrition (*OR* 2.5 [1.8–3.4]).

**Conclusion:**

Under elimination of relevant confounders, there is no significant correlation between sex and risk of SSI after abdominal surgery.

**Supplementary Information:**

The online version contains supplementary material available at 10.1007/s00423-022-02691-6.

## Introduction

Surgical site infections (SSI) rank among the three most frequent healthcare-associated infections in Europe [1]. They account for more than one fifth of all healthcare-associated infections with reported incidence of up to 10% and impose a substantial burden to both health-care systems and patients [1–6]. In the past, multiple patient- and surgery-related factors contributing to the development of SSI were identified including age, body mass index (BMI), and duration of surgery [6–10].

Recently, sex was intensively discussed as a possible risk factor for SSI as several studies reported increased SSI rates for men [11–15]. For instance, two high volume studies analyzing data from German Nosocomial Infections Surveillance reported significantly higher incidence of SSI among men in different procedures including abdominal surgery [11, 12]. However, one study was unadjusted for any confounders [11] and in the other important potential confounders as comorbidities including obesity and malnutrition could not be included into the analysis [12].

Most other studies display unadjusted results or omit some important potential confounders as patient’s age, ASA score, obesity, malnutrition, grade of wound contamination, duration of surgery, and surgical approach (e.g. laparotomy) [11–15]. Therefore, it remains unclear if male sex represents a risk factor for developing SSI after abdominal surgery.

The identification of sex as risk factor for SSI would be important to allow the generation of sex-specific infection control practices in the future. The aim of the present study is to evaluate the impact of sex on surgical site infections after abdominal surgery under elimination of possible confounders.

## Methods

### Trial design, patients, study interventions, and data collection

The study was designed as a multi-center, retrospective analysis of an existing prospective collected database. This database was created in the context of a prospective trial that assessed the effects of structured intraoperative briefings on patient outcomes including SSI [16]. The database includes data from patients undergoing surgery in four Swiss hospitals (2 university hospitals, and two non-university referral centers) between May 2015 and March 2018. The database was initiated after obtaining approval from the respective local ethical committees (leading committee: Kantonale Ethikkommission Bern #161/2014). Written general consent was obtained from individuals of three centers; in the fourth center, the local ethical committee allowed explicitly the inclusion of patients who did not refuse the use of their data. All patients with an indication for elective or emergency abdominal surgery in the participating hospitals were included and entered into an electronic database. Clinicopathological data of 6603 patients including demographics, therapeutic features, surgical procedures, and complications as SSI were extracted. For the final analysis, 82 patients with death within 30 days after surgery were excluded. All surgical treatments were performed by board-certified surgeons. Patients received in-hospital disease surveillance and therapy according to institutional standards and international guidelines. Exclusion criteria were patient age < 18 years, pre-existing SSI, previous surgery at the same surgical site within the past 30 days, procedures without general anesthesia, and proctologic operations. Further patients with American Society of Anaesthesiologists (ASA) score ≥ 5 were excluded.

### Study endpoints

The primary endpoint of this study was defined as SSI within 30 days after surgery according to the Centers for Disease Control and Prevention criteria [17]. Trained study nurses evaluated SSIs in accordance with the Swissnoso SSI surveillance system guide [18]. This guide adheres to the US National Healthcare Safety Network (former National Nosocomial Infection Surveillance, NNIS) standards and includes follow-up interviews by telephone 30 days after surgery [17, 19]. One center entered their perioperative data to the Enhanced Recovery After Surgery Interactive Audit System (ERAS; Encare, Stockholm, Sweden) and SSI were evaluated based on this validated data set [20].

### Statistical analysis

Quantitative and qualitative variables were expressed as mean (standard deviation) and frequency (percentage). For all outcomes, the two groups were compared with the Fisher’s exact test for categorical variables and the Mann–Whitney *U* test for continuous variables, as appropriate. All *p*-values are considered statistically significant if *p* < 0.05. Univariate analysis was applied for patient- and surgery-specific covariates to identify statistically significant association with the occurrence of SSI. Co-variates with statistically significant differences were considered possible confounders for development of SSI. All confounders (*p* < 0.05) were then included in a multivariable regression model in order to assess independency. Subsequently, enter logistic regression was performed. Statistical analysis was performed using SPSS version® 25 (IBM, Armonk, NY, USA).

## Results

A total of 6603 patients undergoing abdominal surgery between May 2015 and March 2018 were analyzed. Mean age and BMI were significantly increased in the SSI group (62.0 vs 56.6 years, *p* < *0.001*; 27.5 vs 26.9 kg/m^2^, *p* = *0.011*). Patient demographics and surgical characteristics are displayed in Table 1. Of the 6603 patients 24.8% underwent colorectal surgery (including small-bowel surgery), 21.4% received hernia repair, 16% underwent cholecystectomy, 11.0% hepato-pancreato-biliary surgery, 10% appendectomy, 8.2% bariatric intervention, 5.6% upper GI (gastrointestinal) surgery, and 3.0% renal or adrenal surgery. Mean duration of surgery (203.9 vs. 121.5 min, *p* < *0.001*), National Nosocomial Infections Surveillance (NNIS) index (1.24 vs. 0.77, *p* < *0.001*), and ASA (2.6 vs. 2.2, *p* < *0.001*) score were significantly higher in the SSI group [21]. The rate of patients with surgery at an university hospital was significantly increased in the SSI group (63.6 vs 53.1%, *p* < 0.001). SSI correlated significantly with increased rates of LOS > 75th percentile (57.0 vs 17.9%, *p* < *0.001*), reoperations (22.7 vs 3.4%, *p* < *0.001*) and mortality (4.6 vs 0.9%, *p* < *0.001*). In 649 of 6603 included patients, SSI was identified (9.8%). Univariate and multivariate analysis of patient and procedure dependent risk factors for SSI are shown in Table 2.

**Table 1:** Patient demographic and surgical characteristics

Variable	SSI (*n* = 649)	No SSI (*n* = 5954)	*p-value*
Age, years, mean years (SD)	62.0 (14.9)	56.6 (17.5)	< *0.001*
BMI, mean kg/m^2^ (SD)	27.5 (6.9)	26.9 (6.5)	*0.011*
Male sex, *n* (%)	399 (61.5)	3367 (56.6)	*0.016*
ASA score, mean (SD)	2.6 (0.7)	2.2 (0.7)	< *0.001*
NNIS index, mean (SD)	1.24 (0.88)	0.77 (0.8)	< *0.001*
Duration of surgery, mean minutes (SD)	203.97 (114.2)	121.55 (88.7)	< *0.001*
Type of surgery, *n* (%)			*0.002*
Colorectal	292 (45.0)	1346 (22.6)	
Hepato-pancreato-biliary	137 (21.1)	592 (9.9)	
Renal and adrenal	6 (0.9)	192 (3.2)	
Appendectomy	23 (3.5)	638 (10.7)	
Cholecystectomy	48 (7.4)	1009 (16.9)	
Hernia	43 (6.6)	1368 (23.0)	
Bariatric	39 (6.0)	503 (8.4)	
Upper GI	61 (9.4)	306 (5.1)	
LOS (> 75%), *n* (%)	369 (57.0)	1063 (17.9)	< *0.001*
Reoperation, *n* (%)	131 (22.7)	185 (3.4)	< *0.001*
30-day mortality, *n* (%)	30 (4.6)	52 (0.9)	< *0.001*

For all italicized values or their subgroup the significance is shown

*ASA*, American Society of Anesthesiology; *BMI*, body mass index; *LOS*, length of stay; *NNIS*, National Nosocomial Infections Surveillance; *SD*, standard deviation; *SSI*, surgical site infection

**Table 2:** Univariate and multivariate analysis of patient and procedure dependent risk factors for SSI

Variable	SSI (*n* = 619)	No SSI (*n* = 5902)	*UV*	MV*	
			*p*	*p*	*OR (95 CI)*
Age higher 65 years, *n* (%)	288 (46.5)	2108 (35.7)	< *0.001*	0.921	1.0 (0.7–1.2)
BMI higher 30 kg/m^2^, *n* (%)	169 (27.8)	1313 (22.7)	*0.004*	< *0.001*	*1.8 (1.3–2.3)*
Male sex, *n* (%)	381 (61.6)	3332 (56.5)	*0.015*	0.290	1.1 (0.8–1.4)
Comorbidities, *n* (%)					
Cancer	310 (50.6)	1695 (29.1)	< *0.001*	*0.018*	*1.3 (1.0*–*1.8)*
Liver cirrhosis	20 (3.7)	123 (2.3)	*0.043*	0.650	0.8 (0.4–1.6)
Diabetes	86 (13.9)	617 (10.5)	*0.009*	0.85	1.0 (0.7–1.4)
Immunosuppression	46 (7.9)	372 (6.5)	0.214		
Chemotherapy	31 (5.0)	159 (2.7)	*0.001*	0.87	0.9 (0.5–1.7)
Alcohol abuse	65 (11.5)	394 (7.2)	< *0.001*	0.38	1.2 (0.8–1.7)
Smoking	171 (27.9)	1509 (26.4)	0.398		
Malnutrition	116 (20.1)	472 (8.8)	< *0.001*	< *0.001*	*2.5 (1.8*–*3.4)*
Perioperative factors, *n* (%)					
Laparotomy	364 (60.1)	2625 (45.1)	< *0.001*	*0.014*	*1.3 (1.0*–*1.7)*
Previous laparotomy	286 (49.2)	2129 (38.6)	< 0.001	*0.007*	1. 4 (1.1–1.7)
Emergency	196 (31.7)	1919 (32.5)	0.667		
Blood transfusion	82 (17.3)	250 (6.5)	< *0.001*	*0.003*	1.7 (1.2–2.4)
NNIS duration > 75^th^ percentile	331 (53.6)	1520 (25.9)	< *0.001*	< *0.001*	*2.3 (1.8*–*2.9)*
NNIS contamination level ≥ 3	189 (31.1)	1104 (19.1)	< *0.001*	*0.045*	*1.3 (1.0*–*1.6)*
NNIS ASA ≥ 3	320 (52.0)	2007 (34.4)	< *0.001*	0.24	1.1 (0.9–1.5)

For all italicized values or their subgroup the significance is shown

^*^Logistic regression multivariate analysis included all variables with *p* < 0.050 in univariate analysis. *ASA*, American Society of Anesthesiology; *BMI*, body mass index; *NNIS*, National Nosocomial Infections Surveillance; *SSI*, surgical site infection

**Fig. 1:**
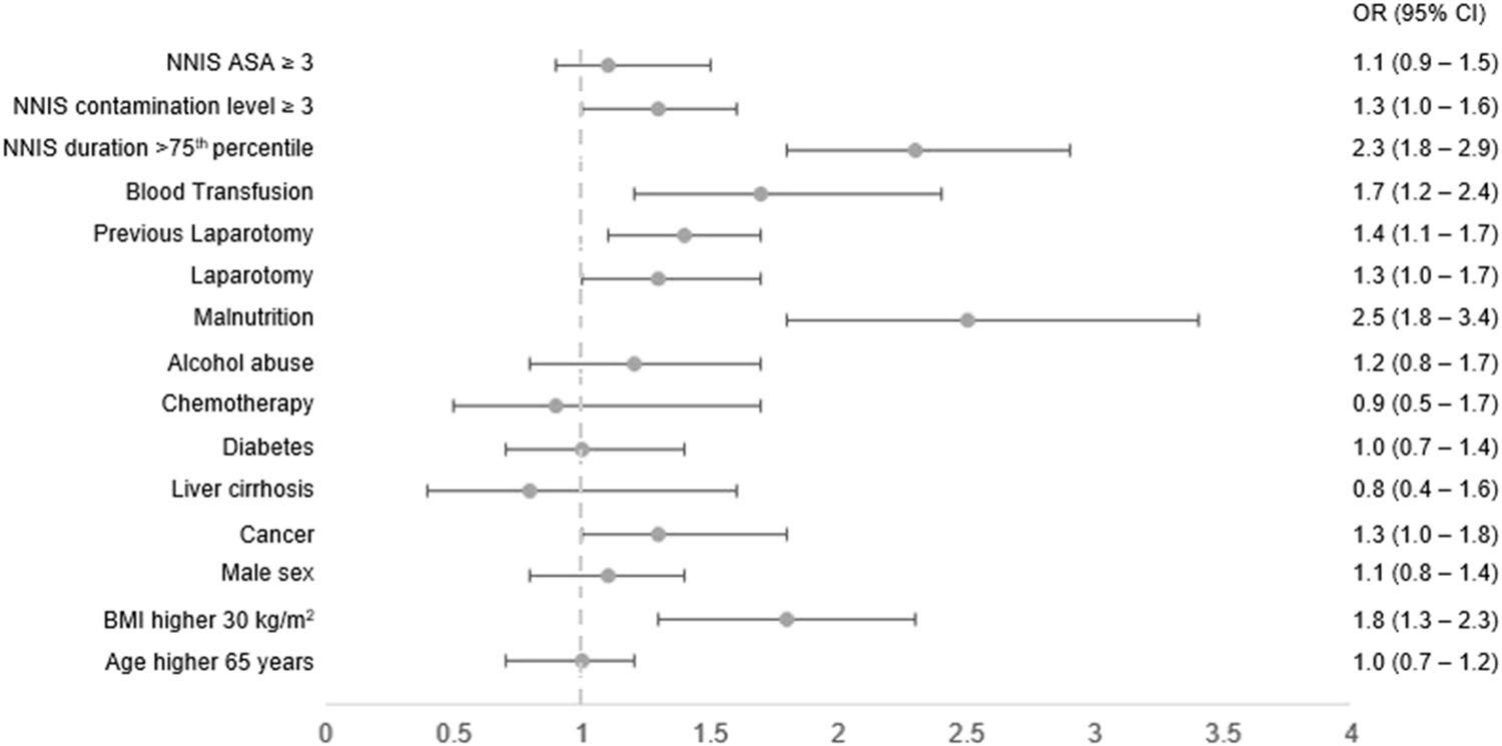
Multivariate analysis of factors associated with SSI following abdominal surgery in the entire study cohort

In univariate analysis, male sex was a significant risk factor for SSI (*p* = *0.015*). Further patient dependent risk factors were age ≥ 65 years (*p* < *0.001*), BMI ≥ 30 kg/m^2^ (*p* = *0.004*), cancer (*p* < *0.001*), liver cirrhosis (*p*= 0.043), diabetes (*p* = *0.009*), chemotherapy (*p* = *0.001*), alcohol abuse (*p* < *0.001*), and malnutrition (*p* < *0.001*). Perioperative factors with significant correlation to SSI were open approach by laparotomy (*p* < *0.001*), previous laparotomy (*p* < *0.001*), type of surgical procedure (*p* = *0.002*), blood transfusion (*p* < *0.001*), and all subsets of the NNIS score (duration > 75th percentile, contamination level ≥ 3, ASA grade ≥ 3, *p* < *0.001*). In multivariate analysis, no association between male sex and risk of SSI (*OR* 1.1 [*CI* 0.8–1.4]) was seen. Figure 1 displays multivariate analysis, where BMI ≥ 30 kg/m^2^ (*OR* 1.8 [*CI* 1.3–2.3]), cancer (*OR* 1.3 [1.0–1.8]), malnutrition (*OR* 2.5 [1.8–3.4]), duration of surgery > 75th percentile (*OR* 2.3 [1.8–2.9]), contamination level ≥ 3 (*OR* 1.3 [1.0–1.6]), laparotomy (*OR* 1.3 [1.0–1.7]), previous laparotomy (*OR* 1.4 [1.1–1.7]), and blood transfusion (*OR* 1.7 [1.2–2.4]) were persistent independent risk factors for SSI. Hosmer–Lemeshow test indicates a good model fit (*p* > 0.05) and the AUC (area under the curve) for the ROC (receiver operating characteristic) curve solid discrimination (ROC curve area = 0.72) for the logistic regression model.

## Discussion

Male sex is controversially discussed as a risk factor for SSI after abdominal surgery. Our study demonstrates no significant correlation between male sex and SSI in abdominal surgery under elimination of multiple relevant confounders.

In contrast to our study, several high volume studies postulate higher risk of SSI for male compared to female patients after abdominal surgery [11–15]. However, the data were either presented completely unadjusted for potential confounders or some important factors, as the duration of surgery, obesity, and malnutrition were not included in the analysis [11–15]. In particular, two studies demonstrating higher risk for SSI for men either displaying unadjusted results [11] or potential confounding through comorbidities as obesity, malnutrition, and wound contamination could not be excluded [12]. Similarly, male sex was identified as independent risk factor for SSI after gastric and pancreatic surgery; however, factors as obesity and malnutrition [14] or wound contamination level [13] were omitted in multivariate analysis. A study addressing the risk of SSI after cholecystectomy, reporting significantly increased risk of SSI for male patients, omitted potential important risk factors as duration of surgery or wound contamination level in the multivariate analysis [15]. The study further demonstrated increased rate of severe biliary disease for men. A possible residual confounding caused by incomplete capture of the increased rate of severe biliary disease in men using claims data is discussed in the study [15].

The studies discussed potential causes for the differences in the SSI rates between male and female patients.[11–13]. Higher rate of visceral fat in men may cause higher complexity of surgery and offer optimal conditions for bacterial growth [12, 13, 22]. Further hormonal or evolutional causes were postulated [11, 12]. However, in all of the named studies, at least one potential important risk factor for SSI as malnutrition, obesity, duration of surgery, or wound contamination level was omitted [11–15]. The potential impact of the missing patient- as well as surgery-related confounders on the postulated higher risk of SSI in these studies therefore remains unknown. [11–15].

In the present study, we demonstrate obesity, malnutrition, cancer, perioperative blood transfusion, prolonged duration of surgery, high contamination level, laparotomy, and previous surgery as independent significant risk factors for SSI. All of the named factors are widely established and show the comparability of our included patients to other centers. The association of obesity and surgical site infection was shown in several studies; the causality seems multifactorial [23]. It is well-known that malnutrition comes along with immune dysfunction, impaired wound-healing, and SSI [24–27]. Increased risk of SSI for patients with cancer may likely be explained due to immunosuppressive state caused by the oncological disease as well as its treatment [28, 29]. The association of prolonged duration of surgery with SSI is consistent with the finding of multiple studies. The effect may be caused by different factors including association with more complex surgery and increased fatigue of the surgical team leading to more technical mistakes [7, 29, 30]. Higher risk of SSI for patients with contaminated or dirty wound levels is commonly known [31]. The significant correlation of blood transfusion with risk of SSI is consistent to several studies [32–35]. On one hand, the need of blood transfusion indicates complex or even uncontrolled intraoperative situation; on the other hand, immunosuppressive effects of blood transfusions are known [33, 36]. Higher rates of SSI for patients with open surgical approach as well as previous laparotomy are likely explained due to association with increased surgical difficulty, more extended surgeries, and higher risk of organ lesion [30, 37–40].

Despite the prospective collection of the data, this study is limited by the retrospective design and therefore was not able to collect all potential sex-associated factors. Therefore, an effect caused by absence of potiental further confounders cannot be excluded. Because of the same reasons, aspects of gender were not included in this study. However, the most relevant strength of the present study is the assessment of a wide variety of potential risk factors, including all of the most commonly established risk factors for development of SSI.

## Conclusion

After adjustment for wide variety of potential confounders including patient-related and procedure-related factors, we could not correlate sex with rate of SSI. However, it is important to incorporate sex in future clinical studies to identify and understand potential effects on surgical outcomes. In particular large, multicentric trials may be relevant in order to correct for potential local confounders. BMI ≥ 30 kg/m^2^, duration of surgery > 75th percentile, high contamination level, surgical approach, cancer, blood transfusion, and malnutrition are significant independent risk factors for SSI in abdominal surgery and should be taken into account to reduce rate of SSI in abdominal surgery in the future.

## Supplementary Information

Below is the link to the electronic supplementary material.

Supplementary file1 (DOCX 15 KB)

## Abbreviations

ASA: American Society of Anesthesiologists
AUC: area under the curve
BMI: Body mass index
GI: gastrointestinal
NNIS: National Nosocomial Infection Surveillance
OR: Odds ratio
ROC: receiver operating characteristic
SSI: Surgical site infection

## References

[1] Suetens C, Latour K, Kärki T, Ricchizzi E, Kinross P, Moro ML (2018). Prevalence of healthcare-associated infections, estimated incidence and composite antimicrobial resistance index in acute care hospitals and long-term care facilities: results from two European point prevalence surveys, 2016 to 2017. Eurosurveillance.

[2] Zarb P, Coignard B, Griskeviciene J, Muller A, Vankerckhoven V, Weist K, Goossens M, Vaerenberg S, Hopkins S, Catry B, Monnet D, Goossens H, Suetens C (2012) National Contact Points for the ECDC pilot point prevalence survey; Hospital Contact Points for the ECDC pilot point prevalence survey. The European Centre for Disease Prevention and Control (ECDC) pilot point prevalence survey of healthcare-associated infections and antimicrobial use. Euro Surveill 17(46):20316. 10.2807/ese.17.46.20316-en 10.2807/ese.17.46.20316-en 23171822

[3] Jenks PJ, Laurent M, McQuarry S, Watkins R (2014). Clinical and economic burden of surgical site infection (SSI) and predicted financial consequences of elimination of SSI from an English hospital. J Hosp Infect.

[4] Badia JM, Casey AL, Petrosillo N, Hudson PM, Mitchell SA, Crosby C (2017). Impact of surgical site infection on healthcare costs and patient outcomes: a systematic review in six European countries. J Hosp Infect.

[5] Global Guidelines for the Prevention of Surgical Site Infection, Geneva: World Health Organization (2018) 3, Important Issues in the Approach to Surgical Site Infection Prevention. Available from: https://www.ncbi.nlm.nih.gov/books/NBK536426/

[6] European Centre for Disease Prevention and Control (2019) Healthcare-associated infections: surgical site infections. In: ECDC. Annual epidemiological report for 2017. ECDC, Stockholm

[7] Cheng H, Chen BP, Soleas IM, Ferko NC, Cameron CG, Hinoul P (2017). Prolonged operative duration increases risk of surgical site infections: a systematic review. Surg Infect.

[8] Gibbons C, Bruce J, Carpenter J, Wilson AP, Wilson J, Pearson A (2011). Identification of risk factors by systematic review and development of risk-adjusted models for surgical site infection. Health technology assessment (Winchester, England).

[9] Korol E, Johnston K, Waser N, Sifakis F, Jafri HS, Lo M (2013). A systematic review of risk factors associated with surgical site infections among surgical patients. PLoS ONE.

[10] Winfield RD, Reese S, Bochicchio K, Mazuski JE, Bochicchio GV (2016). Obesity and the risk for surgical site infection in abdominal surgery. Am Surg.

[11] Langelotz C, Mueller-Rau C, Terziyski S, Rau B, Krannich A, Gastmeier P (2014). Gender-specific differences in surgical site infections: an analysis of 438,050 surgical procedures from the German national nosocomial infections surveillance system. Viszeralmedizin.

[12] Aghdassi SJS, Schröder C, Gastmeier P (2019) Gender-related risk factors for surgical site infections. Results from 10 years of surveillance in Germany. Antimicrob Resist Infect Control 8:95. 10.1186/s13756-019-0547-x 10.1186/s13756-019-0547-x PMC6547551 31171966

[13] Mazmudar A, Vitello D, Chapman M, Tomlinson JS, Bentrem DJ (2017). Gender as a risk factor for adverse intraoperative and postoperative outcomes of elective pancreatectomy. J Surg Oncol.

[14] Kim ES, Kim HB, Song K-H, Kim YK, Kim H-H, Jin HY (2015). Prospective nationwide surveillance of surgical site infections after gastric surgery and risk factor analysis in the Korean nosocomial infections surveillance system (KONIS). Infect Control Hosp Epidemiol.

[15] Warren DK, Nickel KB, Wallace AE, Mines D, Tian F, Symons WJ, Fraser VJ, Olsen MA (2017) Risk factors for surgical site infection after cholecystectomy. Open Forum Infect Dis 4(2):ofx036. 10.1093/ofid/ofx036 10.1093/ofid/ofx036 PMC5419069 28491887

[16] Tschan F, Keller S, Semmer NK, Timm-Holzer E, Zimmermann J, Huber SA (2021). Effects of structured intraoperative briefings on patient outcomes: multicentre before-and-after study. Br J Surg.

[17] ASA Physical Status Classification System (2020) American society of anesthesiologists. https://www.asahq.org/standards-and-guidelines/asa-physical-status-classification-system. Accessed 15 April 2022

[18] Horan TC, Andrus M, Dudeck MA (2008). CDC/NHSN surveillance definition of health care-associated infection and criteria for specific types of infections in the acute care setting. Am J Infect Control.

[19] Kuster SP, Eisenring M-C, Sax H, Troillet N (2017). Structure, process, and outcome quality of surgical site infection surveillance in Switzerland. Infect Control Hosp Epid.

[20] Emori TG, Culver DH, Horan TC, Jarvis WR, White JW, Olson DR (1991). National nosocomial infections surveillance system (NNIS): description of surveillance methods. Am J Infect Control.

[21] Keller S, Grass F, Tschan F, Addor V, Petignat C, Moulin E (2019). Comparison of surveillance of surgical site infections by a national surveillance program and by institutional audit. Surg Infect.

[22] Culver DH, Horan TC, Gaynes RP, Martone WJ, Jarvis WR, Emori TG (1991). Surgical wound infection rates by wound class, operative procedure, and patient risk index. National Nosocomial Infections Surveillance System. Am J Med.

[23] Wajchenberg BL (2000). Subcutaneous and visceral adipose tissue: their relation to the metabolic syndrome. Endocr Rev.

[24] Thelwall S, Harrington P, Sheridan E, Lamagni T (2015). Impact of obesity on the risk of wound infection following surgery: results from a nationwide prospective multicentre cohort study in England. Clin Microbiol Infect.

[25] Bourke CD, Berkley JA, Prendergast AJ (2016). Immune dysfunction as a cause and consequence of malnutrition. Trends Immunol.

[26] Skeie E, Koch AM, Harthug S, Fosse U, Sygnestveit K, Nilsen RM (2018). A positive association between nutritional risk and the incidence of surgical site infections a hospital-based register study. PloS one.

[27] Hennessey DB, Burke JP, Ni-Dhonochu T, Shields C, Winter DC, Mealy K (2010). Preoperative hypoalbuminemia is an independent risk factor for the development of surgical site infection following gastrointestinal surgery: a multi-institutional study. Ann Surg.

[28] Stechmiller JK (2010). Understanding the role of nutrition and wound healing. Nutr Clin Pract.

[29] Payne WG, Naidu DK, Wheeler CK, Barkoe D, Mentis M, Salas RE (2008). Wound healing in patients with cancer. Eplasty.

[30] Cheng K, Li J, Kong Q, Wang C, Ye N, Xia G (2015). Risk factors for surgical site infection in a teaching hospital: a prospective study of 1,138 patients. Patient Prefer Adherence.

[31] Kurmann A, Vorburger SA, Candinas D, Beldi G (2011). Operation time and body mass index are significant risk factors for surgical site infection in laparoscopic sigmoid resection: a multicenter study. Surg Endosc.

[32] Ortega G, Rhee DS, Papandria DJ, Yang J, Ibrahim AM, Shore AD (2012). An evaluation of surgical site infections by wound classification system using the ACS-NSQIP. J Surg Res.

[33] Higgins RM, Helm MC, Kindel TL, Gould JC (2019). Perioperative blood transfusion increases risk of surgical site infection after bariatric surgery. Surg Obes Relat Dis.

[34] Fawley J, Chelius TH, Arca MJ (2018). Relationship between perioperative blood transfusion and surgical site infections in pediatric general and thoracic surgical patients. J Pediatr Surg.

[35] Helm M, Gould J, Higgins R (2017). A502 - perioperative blood transfusion increases risk of surgical site infection following bariatric surgery. Surg for Obesity and Related Dis.

[36] Dosch AR, Grigorian A, Delaplain PT, Bartholomew TS, Won EJ, Gabriel V (2019). Perioperative blood transfusion is associated with an increased risk for post-surgical infection following pancreaticoduodenectomy. HPB (Oxford).

[37] Youssef LA, Spitalnik SL (2017). Transfusion-related immunomodulation: a reappraisal. Curr Opin Hematol.

[38] Alkaaki A, Al-Radi OO, Khoja A, Alnawawi A, Alnawawi A, Maghrabi A (2019). Surgical site infection following abdominal surgery: a prospective cohort study. Can J Surg.

[39] Li Z, Li H, Lv P, Peng X, Wu C, Ren J (2021). Prospective multicenter study on the incidence of surgical site infection after emergency abdominal surgery in China. Sci Rep.

[40] Fahrner R, Malinka T, Klasen J, Candinas D, Beldi G (2014). Additional surgical procedure is a risk factor for surgical site infections after laparoscopic cholecystectomy. Langenbecks Arch Surg.

[41] Zwicky SN, Stroka D, Zindel J (2021) Sterile Injury Repair and Adhesion Formation at Serosal Surfaces. Front Immunol 12:684967. 10.3389/fimmu.2021.684967 10.3389/fimmu.2021.684967 PMC8160448 34054877

