# Supplementary material for: No impact of sex on surgical site infections in abdominal surgery: a multi-center study
Source: Langenbecks Arch Surg. 2022 Oct 10;407(8):3763–9. doi: 10.1007/s00423-022-02691-6 (PMC9722878; doi:10.1007/s00423-022-02691-6)
Supplement: Supplementary file 1 — Supplementary file1 (DOCX 15 KB) [file 423_2022_2691_MOESM1_ESM.docx]

**Table for review 1.** Patient demographic and surgical characteristics for male and female patients.

| **Variable** | **Male**  **(n=3766)** | **Female**  **(n=2837)** | ***p-value*** |
| --- | --- | --- | --- |
| Age, years, mean years (SD) | 58.0 (16.6) | 55.7 (18.3) | *<0.001* |
| BMI, mean kg/m^2^ (SD) | 26.8 (5.7) | 27.2 (7.5) | *0.034* |
| ASA Score, mean (SD) | 2.3 (0.7) | 2.2 (0.7) | *<0.001* |
| Comorbidities, n (%) |  |  |  |
| Cancer | 1283 (34.5) | 771 (27.5) | *<0.001* |
| Liver cirrhosis | 106 (3.1) | 42 (1.7) | *0.001* |
| Diabetes | 478 (12.7) | 238 (8.4) | *<0.001* |
| Immunosuppression | 262 (7.2) | 168 (6.2) | *0.116* |
| Chemotherapy | 124 (3.3) | 75 (2.7) | *0.120* |
| Alcohol abuse | 367 (10.4) | 107 (4.1) | *<0.001* |
| Smoking | 1054 (28.6) | 647 (23.7) | *<0.001* |
| Malnutrition | 352 (10.1) | 271 (10.6) | *0.532* |
| Duration of surgery, mean minutes (SD) | 134.8 (99.7) | 122.8 (87.2) | *<0.001* |
| Type of surgery, n (%) |  |  |  |
| Colorectal | 933 (24.8) | 705 (24.9) | 0.954 |
| Hepato-Pancreato-Biliary | 412 (10.9) | 317 (11.2) | 0.781 |
| Renal and Adrenal | 104 (2.8) | 94 (3.3) | 0.215 |
| Appendectomy | 359 (9.5) | 302 (10.6) | 0.136 |
| Cholecystectomy | 481 (12.8) | 576 (20.3) | *0.001* |
| Hernia | 1046 (27.8) | 365 (12.9) | *<0.001* |
| Bariatric | 199 (5.3) | 343 (12.1) | *<0.001* |
| Upper GI | 232 (6.2) | 135 (4.8) | *0.015* |
| Perioperative Factors, n (%) |  |  |  |
| Laparotomy | 1754 (47.2) | 1279 (45.8) | *0.268* |
| Previous Laparotomy | 1246 (35.2) | 1212 (46.0) | *<0.001* |
| Emergency | 1186 (31.5) | 979 (34.5) | *0.010* |
| Blood Transfusion | 236 (9.5) | 117 (6.2) | *<0.001* |
| NNIS duration >75^th^ percentile | 1140 (30.4) | 736 (26.1) | *<0.001* |
| NNIS contamination level ≥ 3 | 802 (21.7) | 531 (19.1) | *0.009* |
| NNIS ASA ≥ 3 | 1468 (39.4) | 932 (33.2) | *<0.001* |
| Outcome parameters, n (%) |  |  |  |
| SSI | 399 (10.6) | 250 (8.8) | *0.016* |
| LOS (>75%) | 834 (22.2) | 598 (21.1) | *0.300* |
| Reoperation | 211 (6.0) | 105 (4.1) | *0.001* |
| 30-Day mortality | 53 (1.4) | 29 (1.0) | 0.162 |
